# Surviving the Shocks: A Contemporary of Short-coupled Ventricular Fibrillation

**DOI:** 10.19102/icrm.2024.15033

**Published:** 2024-03-15

**Authors:** Deepti Ranganathan, Eduardo Sanhueza Gonzalez, Maria Terricabras, Christopher C. Cheung

**Affiliations:** 1 Division of Cardiology, Sunnybrook Health Science Centre, Toronto, Ontario, Canada; 2 Queen’s University, Kingston Health Science Centre, Kingston, Ontario, Canada

**Keywords:** Idiopathic ventricular fibrillation, cardiac arrest, moderator band PVC

## Abstract

A young man presented following successful cardiac resuscitation after an out-of-hospital cardiac arrest. During his admission, he had multiple runs of short-coupled ventricular fibrillation with a similar morphology premature ventricular complex (PVC) trigger. He was brought to the electrophysiology laboratory, and, with a high dose of isoprenaline, the PVC was localised to the moderator band. Ablation induced short runs of ventricular tachycardia before elimination of the PVC. He subsequently underwent subcutaneous implantable cardiac defibrillator implantation before his discharge.

## Case presentation

A young man with two prior out-of-hospital cardiac arrests presented following a third out-of-hospital cardiac arrest and resuscitation. He had experienced two prior ventricular fibrillation (VF) arrests 18 months prior during sympathomimetic drug use. Despite counseling, the patient had previously declined to receive an implantable cardioverter-defibrillator (ICD) but was amenable to taking metoprolol following the second cardiac arrest in conjunction with refrainment from sympathomimetic drug use.

His past medical history included a childhood history of ventricular septal defect and patent ductus arteriosus (both closed spontaneously). He had no family history of sudden death. He was on lisdexamfetamine for attention-deficit hyperactivity disorder, which was discontinued after his second cardiac arrest, and he was commenced on a β-blocker, which he was not taking regularly before this presentation.

**Figure 1:**
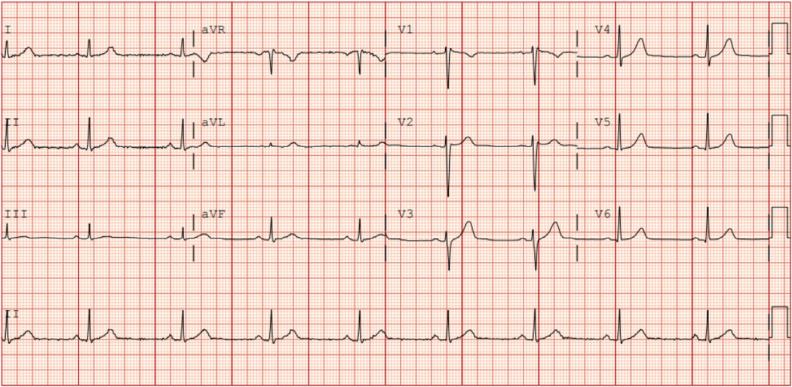
Baseline electrocardiogram recorded following the patient’s third aborted cardiac arrest.

Results of initial blood tests, including for electrolytes, and toxicology screening findings were all negative, but he tested positive for influenza A. Comprehensive investigations included a repeat 12-lead electrocardiogram (ECG) demonstrating normal sinus rhythm **(Figure 1)**, an unremarkable cardiac magnetic resonance imaging scan, a negative procainamide provocation test result, and negative genetic testing results (broad arrhythmia panel).

**Figure 2:**
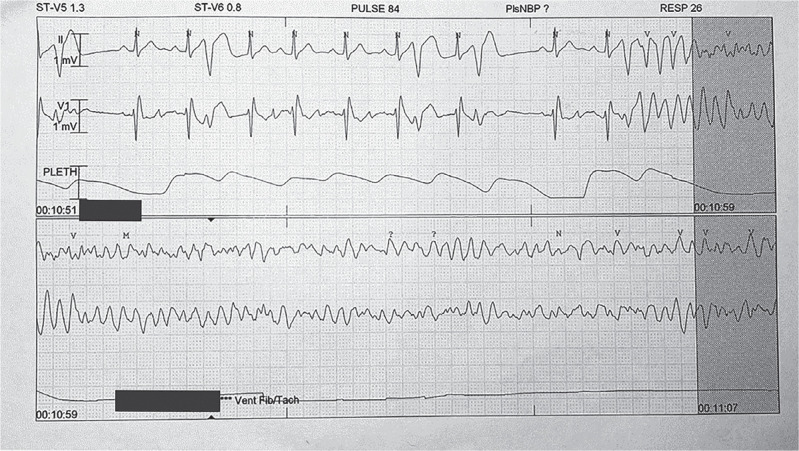
The first-documented instance of ventricular fibrillation occurred while the patient was an inpatient, with short-coupled premature ventricular contractions found to have initiated ventricular fibrillation.

**Figure 3:**
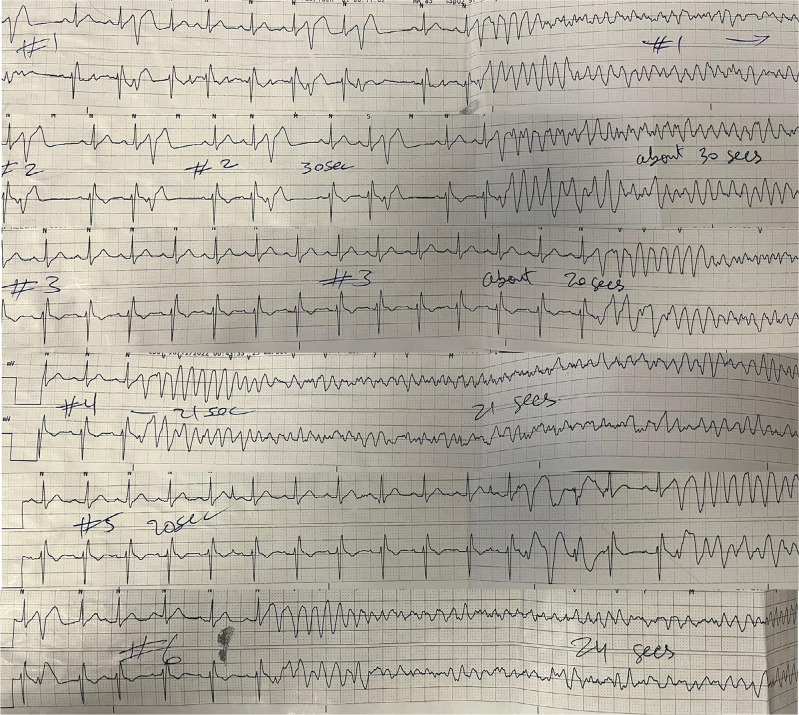
Further episodes of ventricular fibrillation were all preceded by a similar morphology of short-coupled premature ventricular contractions found to have initiated ventricular fibrillation.

After initial resuscitations, the patient had a further six recurrent episodes of VF in the intensive care unit, all of which had a similar-morphology short-coupled PVC trigger **(Figures 2 and 3)**.

**Figure 4:**
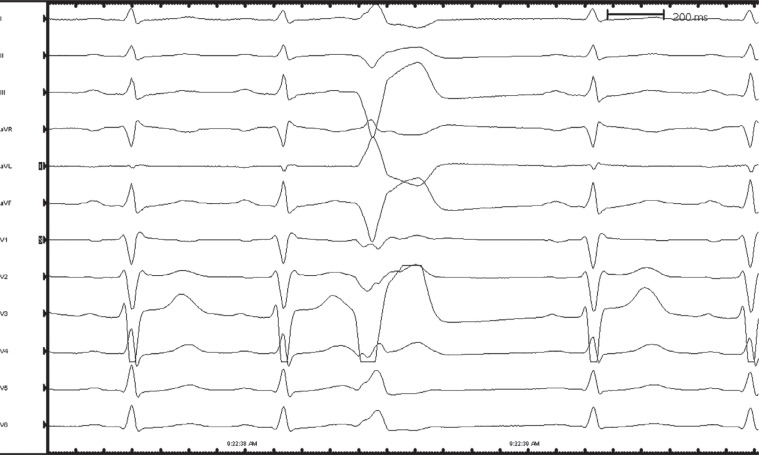
Premature ventricular contraction induced during an electrophysiology study, on a high dose of isoproterenol, showing a left bundle branch block pattern, V_4_ transition, and superiorly directed axis, likely arising from the moderator band.

**Figure 5:**
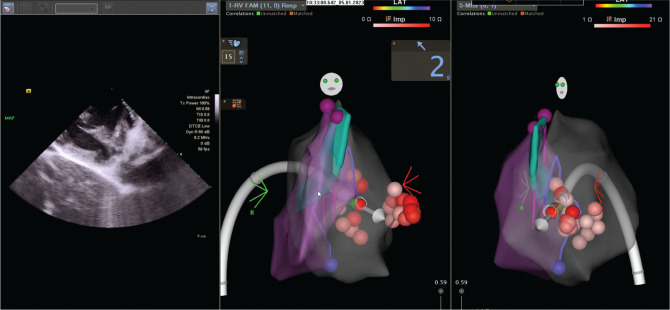
Intracardiac echocardiography image and electroanatomic map showing the ablation catheter in contact with the moderator band.

Given the patient’s recurrent episodes of VF with an apparent uniform-morphology PVC trigger based on the telemetry findings, the patient was brought to the electrophysiology laboratory for further investigation. High-dose isoproterenol-induced PVCs with a left bundle branch block pattern, V_4_ transition, and superiorly directed axis, likely arising from the moderator band (MB), were noted **(Figure 4)**. Activation and pace mapping were performed to localize the ablation site, and intracardiac echocardiography (ICE) confirmed catheter positioning and contact with the MB **(Figure 5)**. Initial ablation induced short runs of ventricular tachycardia, before termination and elimination of the PVC. The patient also underwent subcutaneous ICD implantation before discharge, and quinidine medical therapy was recommended. The patient did not experience any recurrent events during 9 months of follow-up.

## Discussion

Idiopathic VF (IVF) occurs in the absence of structural heart disease and accounts for 5%–7% of survivors of unexplained cardiac arrest.^1^ Short-coupled VF (SCVF) is an entity that has gained focus as a distinct primary electrical disorder, with a PVC coupling interval of <350 ms acting as a trigger for VF.^2^ The 2013 Heart Rhythm Society/European Heart Rhythm Association/Asia Pacific Heart Rhythm Society Expert Consensus Statement on the Diagnosis and Management of Patients with Inherited Primary Arrhythmia Syndromes recommends both anti-arrhythmic therapy with quinidine and ablation of Purkinje potentials in patients with uniform-morphology PVCs with a Class IIb recommendation.^3^ The use of quinidine, a potent Na_v_1.5 inhibitor, is shown to be highly effective in the management of IVF and should be considered in patients with recurrent events.^4^ However, in drug-refractory cases of IVF, ablation of PVCs triggering VF can be considered.^5^

In this case, the PVC morphology had a left bundle branch block superior axis with V_4_ transition suggestive of an MB-PVC with a basal breakout as opposed to the more commonly seen apical exit. The MB is a pro-arrhythmic structure, giving rise to the right Purkinje system, and MB-PVCs have been reportedly associated with IVF.^6^ Recent reports have described two excitable yet uncoupled components, the RV myocardium and the Purkinje fibers, that are electrically compartmentalized and provide a substrate for macro–re-entry.^7^ In cases of right ventricular PVCs, a diagnosis of arrhythmogenic right ventricular dysplasia/cardiomyopathy (ARVD/C) should be considered, as PVCs due to ARVD/C can similarly initiate ventricular tachyarrhythmias, re.0sulting in sudden cardiac arrest. However, an unremarkable cardiac magnetic resonance imaging scan, negative genetic testing results with a broad arrhythmia panel, and repeated short-coupled VF episodes excluded this diagnosis in our patient.

Given the recurrent episodes of PVCs inducing VF in our case, we performed catheter ablation to target the MB-PVCs. In a study by Knecht et al. reporting the long-term follow-up of 38 patients undergoing catheter ablation of IVF, 18% of patients experienced recurrent VF with a median time to recurrence of 4 months.^8^ Ablation significantly reduced the number of events, with 36 out of 38 patients remaining free from VF over a mean of 52 ± 28 months of follow-up. Accurate visualization and stable contact are critical to ablation success, given the thick and mobile nature of the MB. Finally, catheter ablation should be used as an adjunct to anti-arrhythmic therapy (ie, quinidine) and ICD implantation. Our patient remains well and has experienced no recurrence of VF to date over an extended follow-up period.

## References

[1] Conte G, Belhassen B, Lambiase P (2019). Out-of-hospital cardiac arrest due to idiopathic ventricular fibrillation in patients with normal electrocardiograms: results from a multicentre long-term registry. Europace.

[2] Steinberg C, Davies B, Mellor G (2021). Short-coupled ventricular fibrillation represents a distinct phenotype among latent causes of unexplained cardiac arrest: a report from the CASPER registry. Eur Heart J.

[3] Priori SG, Wilde AA, Horie M (2013). HRS/EHRA/APHRS expert consensus statement on the diagnosis and management of patients with inherited primary arrhythmia syndromes: document endorsed by HRS, EHRA, and APHRS in May 2013 and by ACCF, AHA, PACES, and AEPC in June 2013. Heart Rhythm.

[4] Malhi N, Cheung CC, Deif B (2019). Challenge and impact of quinidine access in sudden death syndromes: a national experience. JACC Clin Electrophysiol.

[5] Haïssaguerre M, Shoda M, Jaïs P (2002). Mapping and ablation of idiopathic ventricular fibrillation. Circulation.

[6] Haïssaguerre M, Vigmond E, Stuyvers B, Hocini M, Bernus O (2016). Ventricular arrhythmias and the His-Purkinje system. Nat Rev Cardiol.

[7] Sadek MM, Benhayon D, Sureddi R (2015). Idiopathic ventricular arrhythmias originating from the moderator band: electrocardiographic characteristics and treatment by catheter ablation. Heart Rhythm.

[8] Knecht S, Sacher F, Wright M (2009). Long-term follow-up of idiopathic ventricular fibrillation ablation: a multicenter study. J Am Coll Cardiol.

